# Altered regional neural activity and functional connectivity in patients with non-communicating hydrocephalus: a resting-state functional magnetic resonance imaging study

**DOI:** 10.3389/fneur.2024.1438149

**Published:** 2024-08-14

**Authors:** Xiaoyuan Huang, Lu Jin, Tengwu Chang, Jian Liu, Yuan Qu, Jinyong Li, Wenju Bai, Chuzhong Li, Jichao Wang

**Affiliations:** ^1^Graduate School, Xinjiang Medical University, Ürümqi, China; ^2^Department of Neurosurgery, Beijing Tiantan Hospital, Capital Medical University, Beijing, China; ^3^Department of Neurosurgery, People’s Hospital of Xinjiang Uygur Autonomous Region, Ürümqi, China; ^4^Department of Orthopaedics, People’s Hospital of Xinjiang Uygur Autonomous Region, Ürümqi, China; ^5^Radiographic Image Center, People’s Hospital of Xinjiang Uygur Autonomous Region, Ürümqi, China; ^6^Beijing Neurosurgical Institute, Capital Medical University, Beijing, China

**Keywords:** non-communicating hydrocephalus, regional homogeneity, degree centrality, functional connectivity, cognitive impairment

## Abstract

**Introduction:**

Cognitive impairment is a frequent clinical symptom of non-communicating hydrocephalus (NCH) involving multiple domains, including executive function, working memory, visual-spatial function, language, and attention. Functional magnetic resonance imaging (fMRI) can be used to obtain information on functional activity in local brain areas and functional connectivity (FC) across multiple brain regions. However, studies on the associated cognitive impairment are limited; further, the pathophysiological mechanisms of NCH with cognitive impairment remain unclear. Here, we aimed to explore alterations in regional neural activity and FC, as well as the mechanisms of cognitive impairment, in patients with NCH.

**Methods:**

Overall, 16 patients with NCH and 25 demographically matched healthy controls (HCs) were assessed using the Mini-Mental State Examination (MMSE) and fMRI. Changes in regional homogeneity (ReHo), degree centrality (DC), and region of interest-based FC were analyzed in both groups. The relationship between fMRI metrics (ReHo, DC, and FC) and MMSE scores in patients with NCH was also investigated.

**Results and discussion:**

Compared with the HC group, the NCH group exhibited significantly lower ReHo values in the left precentral and postcentral gyri, and significantly higher ReHo values in the left medial prefrontal cortex (MPFC). The NCH group also showed significantly higher DC values in the bilateral MPFC compared with the HC group. Regarding seed-based FC, the MPFC showed reduced FC values in the right superior parietal and postcentral gyrus in the NCH group compared with those in the HC group. Moreover, within the NCH group, MMSE scores were significantly negatively correlated with the ReHo value in the left MPFC and the DC value in the bilateral MPFC, whereas MMSE scores were significantly positively correlated with FC values. To conclude, regional neural activity and FC are altered in patients with NCH and are correlated with cognitive impairment. These results advance our understanding of the pathophysiological mechanisms underlying the association between NCH and cognitive impairment.

## 1 Introduction

Hydrocephalus is a neurological disorder characterized by an increased volume of cerebrospinal fluid (CSF). This leads to ventricular swelling, which exerts pressure on the brain and skull, causing extensive damage to neural structures (1). The etiologies of hydrocephalus include hypersecretion, circulation disturbance (non-communicating hydrocephalus, NCH), malabsorption (communicating hydrocephalus), and other specific types of unknown etiologies such as idiopathic normal pressure hydrocephalus (iNPH) (2). NCH, also known as obstructive hydrocephalus, is typically caused by a variety of pathologies in adults, including cerebral hemorrhage, tumors, cerebral trauma, and intracranial infections (3, 4). It usually presents with symptoms such as headaches, vision disturbances, cognitive impairment, motor dysfunction, and incontinence (5). Cognitive impairment is a frequent clinical symptom of NCH involving multiple domains, including executive function, working memory, visual-spatial function, language, and attention (6). A study on neuropsychological deficits in patients with NCH and aqueductal stenosis revealed that cognitive impairment in hydrocephalus is linked to fornix damage and frontal dysfunction (7). Moreover, cognitive impairment in iNPH is associated with aberrant CSF dynamics, frontostriatal and entorhinal-hippocampal circuit dysfunction, and neuromodulation abnormalities (8–10). Despite extensive research on the neuropathological mechanisms underlying hydrocephalus, there have been comparatively few studies on the associated cognitive impairment, and the pathophysiological mechanisms of NCH with cognitive impairment remain unclear.

Functional magnetic resonance imaging (fMRI) can be used to obtain information on functional activity in local brain areas and functional connectivity (FC) across multiple brain regions. This can then be analyzed using functional separation and integration methods (11). Regional homogeneity (ReHo) is a commonly used measure in the former category; it is a voxel-based measure that evaluates the similarity between the time series of a given voxel and its nearest neighbors, as calculated by the Kendall coefficient of concordance of the BOLD time series. The higher the ReHo value, the greater the coherence and centrality of activity in the brain region (12). ReHo has been extensively explored as a parameter underlying neuropsychiatric diseases, including cognitive impairment, consciousness impairment, and Parkinson’s disease (13–15).

Degree centrality (DC) and region of interest (ROI)-based FC analyses are the commonly used functional integration methods. DC is a method driven by data at the voxel level that evaluates the connectivity strength between every voxel and identifies important functional hubs within the brain (16). In the absence of prior assumptions, DC can be used to study functional changes across the entire brain (17), although it does not indicate which regions are connected (18). ROI-based FC also referred to as seed-based FC, determines the seed regions based on assumptions or prior information. It calculates the correlations of time series between ROIs and other brain regions (18, 19). In other words, ROI-based FC can provide comprehensive and specific information on brain FC and help further understand the pathophysiological changes in NCH with cognitive impairment when combined with ReHo and DC methods.

Resting-state fMRI has been used recently to investigate the mechanisms of cognitive impairment in hydrocephalus, providing unique insights into neural activity and connectivity alterations. For example, several neuroimaging studies (20–22) have shown that cognitive and executive function impairments in iNPH and infantile hydrocephalus are associated with alterations in default mode network (DMN) connectivity. However, no studies have explored the changes in regional neural activity and FC, nor the mechanisms underlying cognitive impairment, in patients with NCH.

Therefore, this study aimed to explore changes in regional neural activity and FC, as well as the mechanisms underlying cognitive impairment in patients with NCH. Specifically, the study objectives were to (1) perform ReHo and DC analyses to inspect the functional brain activities of patients with NCH; (2) investigate the correlations between anomalous REHO and DC values and the severity of cognitive impairment in these patients; (3) conduct FC analyses based on regions that showed altered ReHo and DC values, as well as DMN core nodes, to explore possible functional dysconnectivity in patients with NCH and its association with cognitive impairment.

## 2 Materials and methods

### 2.1 Participants

In total, 16 patients with NCH and 25 healthy controls (HCs) were enrolled in the study. The two groups were matched for age, sex, and educational levels. All participants met the following criteria: (1) Enlargement of the ventricular system (Evans’ Index >0.3). (2) Compression of the fourth ventricle, midbrain aqueduct, or third ventricle by adjacent lesions, resulting in the obstruction of CSF circulation. (3) No lesions observed in the gray matter or white matter of either hemisphere. (4) No history of craniocerebral surgery or mental and psychological disorders. (5) No medical history of conditions that could contribute to cognitive impairment. Datasets presenting MRI image distortion and excessive head motion were excluded. Two patients were excluded owing to excessive head motion.

### 2.2 Clinical assessments

Clinical evaluations were performed by a surgeon experienced in neuropsychology. All participants were assessed on the day of the MRI. Specifically, the Mini-Mental State Examination (MMSE) (23) was administered to evaluate general cognitive ability, with lower scores indicating poorer cognitive function. Detailed records of age, sex, education, and handedness were maintained for all participants.

### 2.3 Standard protocol approval and consent

This study was approved by the Ethics Committees of the Beijing Tiantan Hospital and the People’s Hospital of the Xinjiang Uygur Autonomous Region. All the participants provided written informed consent.

### 2.4 MRI data acquisition

All subjects were scanned using a 3.0 Tesla Siemens scanner (Siemens Healthineers, Erlangen, Germany) with a standard head coil. For rs-fMRI images, axial slices were obtained in a single-shot gradient echo-planar imaging (EPI) sequence for each subject (30 axial slices, acquisition matrix = 64 × 64, slice thickness/gap = 5/0.5 mm, repetition time = 2,000 msec, echo time = 30 msec, and FOV = 192 × 192 mm^2^). The 3D T1-weighted sagittal anatomical image was acquired with a magnetism-prepared rapid acquired with gradient echo (MPRAGE) sequence: (192 slices, slice thickness/gap = 1/0 mm, acquisition matrix = 256 × 256, fip angle = 8 deg, TI/TR/TE = 900/2,300/2.3 msec, FOV = 256 × 256 mm^2^). Throughout the scan, each participant was instructed to unwind, close their eyes, and refrain from thinking.

### 2.5 Image preprocessing

The rs-fMRI data were preprocessed using Statistical Parametric Mapping (SPM12, http://www.fl.ion.ucl.ac.uk/spm) (24) and the resting-state fMRI data analysis toolkit (RESTplus v1.28, http://www.restfmri.net) (25). The first 10 volumes were discarded for each subject to avoid transient signal changes. Then, slice-timing adjustment and realignment for head motion correction were carried out. The study excluded participants who had a head motion larger than 3.0 mm or a rotation higher than 3.0° in the *x*, *y*, or *z* directions. The data underwent spatial standardization according to the Montreal Neurological Institute (MNI) template (resampling voxel size of 3 × 3 × 3 mm^3^). To improve the signal-to-noise ratio, spatial smoothing with a Gaussian kernel of 6 × 6 × 6 mm full-width at half-maximum (FWHM) was performed before calculating DC and ReHo measures, but not for FC analysis. Detrending was used to eliminate linear trends from the normalized time series, and a number of nuisance signals were regressed out, such as white matter, CSF, and Friston’s 24-head motion parameter (26). Global signal regression was not performed because this procedure remains controversial. Finally, for every time course, bandpass filtering (0.01–0.08 Hz) was carried out.

### 2.6 ReHo analysis

ReHo calculation was performed in RESTplus. It was calculated based on Kendall’s coefficient of concordance (KCC-ReHo) of the time series of a given voxel’s signal time course and its 26 nearest neighboring voxels (27). Finally, the ReHo maps were *Z*-standardized and spatial smoothed.

### 2.7 DC analysis

DC was performed using RESTplus software. For each voxel, the bold time process is extracted and associated with every voxel in the brain. The correlation of each time course between a gray matter mask voxel and every other voxel in the brain was calculated to create Pearson’s correlation coefficient matrix. A threshold of *r* > 0.25 was established for Pearson’s correlation coefficient. Then, the correlation matrix was binarized for further statistical analysis. Afterward, Fisher’s *Z*-standardization was performed on DC maps.

### 2.8 FC analysis

**Figure 1:**
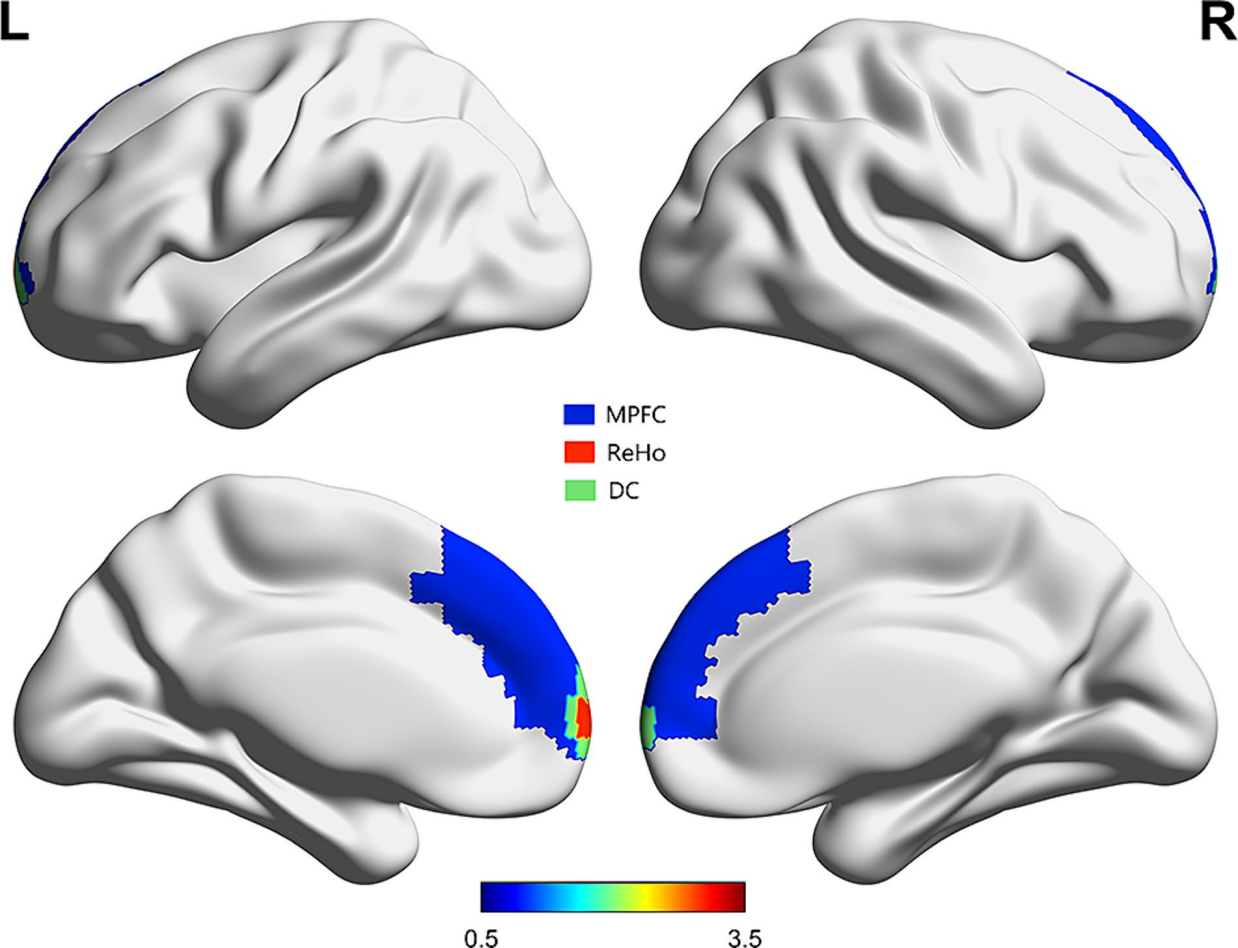
Regions of ReHo and DC which correlated with MMSE scores overlaid on MPFC [from automated anatomical labelling atlas (31)].

To identify the DMN FC map, two core nodes of the DMN were selected as ROIs: the medial prefrontal cortex (MPFC) and the posterior cingulate cortex (PCC)-precuneus. The main hubs of the DMN include the MPFC, PCC, precuneus, and lateral parietal cortex (28). MPFC and PCC/precuneus are commonly used as seed regions in studies investigating DMN’s role in cognition (29). These ROIs were chosen based on prior studies (30). Both areas were defined using masks from the Automated Anatomical Labelling atlas (AAL3v1; http://www.gin.cnrs.fr/tools/aal-aal3) (Figure 1) (31). Subsequently, FC maps were generated by obtaining the ROI time courses, computing the average BOLD time series for all voxels within each ROI, and calculating Pearson’s correlation coefficients between the ROIs and the time courses of every voxel in the whole brain. Finally, Fisher’s *Z*-standardization was applied to the FC maps.

### 2.9 Statistical analysis

Sociodemographic and clinical information were analyzed with GraphPad Prism version 9.5.0. The Shapiro–Wilk test is utilized to evaluate whether the data conforms to a normal distribution. We employed a two-sample t-test to analyze normally distributed variables, and non-parametric tests, including the Mann–Whitney *U*-test and chi-square test, were utilized for non-normally distributed data. Statistical significance was defined as *p* < 0.05. General linear model analysis in SPM12 was used to compute and analyze the rs-fMRI metrics between the two groups. Age, education level, sex, and mean FD were included as nuisance covariates in two-sample *t*-tests to investigate differences in ReHo, DC, and FC between the groups. Following false discovery rate (FDR) correction, cluster-level *p* < 0.05 and voxel-wise *p* < 0.001 were considered statistically significant. The AAL atlas was used to localize the suprathreshold results. Spearman’s correlation analysis was employed to investigate the correlation between fMRI metrics (ReHo, DC, and FC) and MMSE scores in patients with NCH, with statistical significance set at *p* < 0.05. Brain maps and 3D brain models were displayed using the Data Processing & Analysis of Brain Imaging toolbox (DPABI, http://www.restfmri.net/) (32) and BrainNet viewer (33).

## 3 Results

### 3.1 Sociodemographic and clinical characteristics

There were no significant differences in age, sex, and education between the two groups. However, the MMSE scores were significantly different (*p* < 0.001). Head movement also differed significantly between the two groups (*p* = 0.0348). Consequently, the mean FD value was included as a covariate in the analysis of all metrics. The sociodemographic characteristics and clinical information are presented in Table 1.

**Table 1:** Sociodemographic and clinical characteristics of study participants.

Characteristics	NCH (*n* = 16)	HCs (*n* = 25)	Group comparison *p*-values
Age (mean ± SD, years)	45.50 ± 11.2	42.56 ± 14.04	0.3661^a^
Sex (male/female)	8/8	16/9	0.5178^b^
Education (mean ± SD, years)	11.94 ± 3.79	13.64 ± 3.85	0.2412^a^
Handedness (L/R/B)	15R/1B	23R/2B	—
MMSE (mean ± SD)	27.13 ± 1.408	29.52 ± 1.046	<0.001^c^^,^^d^
Mean FD (mean ± SD)	0.20 ± 0.11	0.14 ± 0.05	0.0348^c^^,^^d^

NCH, non-communicating hydrocephalus; HCs, healthy controls; SD, standard deviation.

a Two-sample *t*-test.

b Chi-square test.

c Mann–Whitney *U* test.

d *p* < 0.05, significant difference between groups.

### 3.2 ReHo and correlation analysis

**Figure 2:**
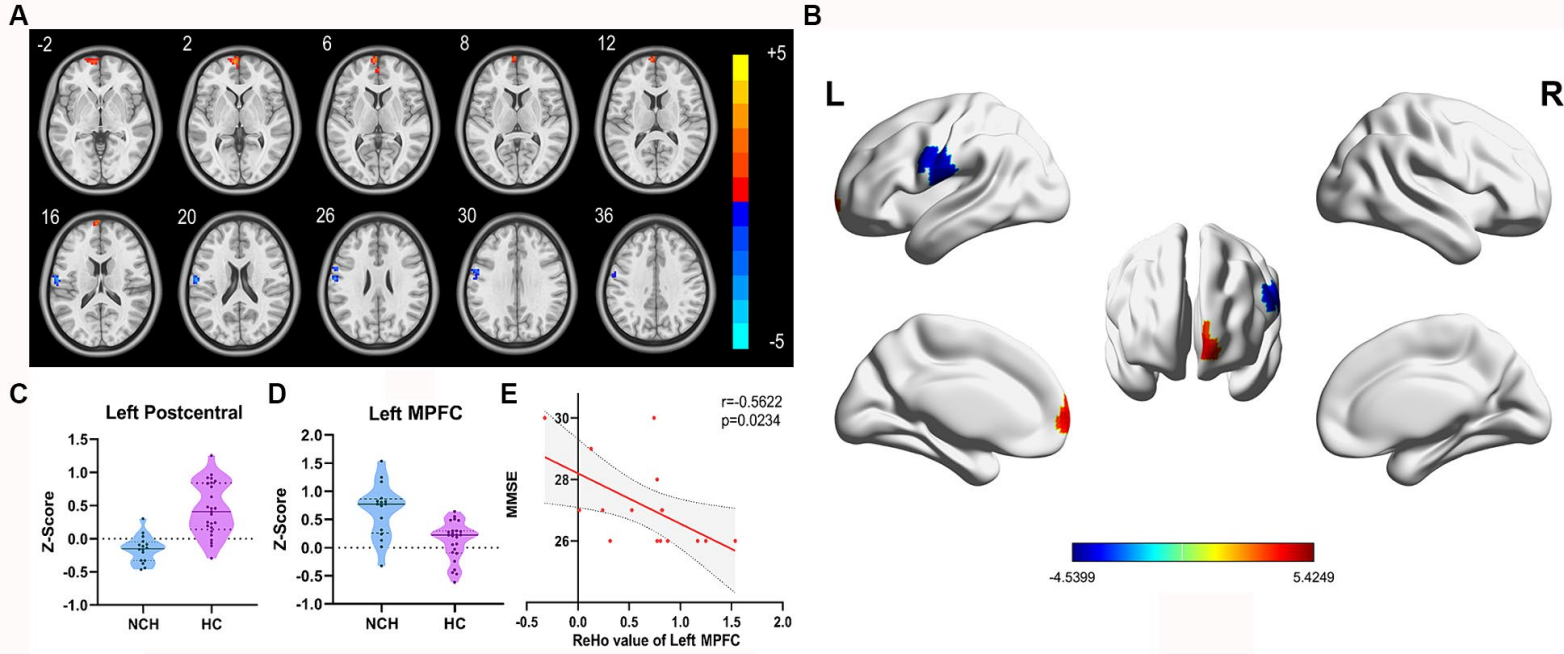
Significantly different ReHo map and the correlation between MMSE scores and ReHo values of left MPFC. **(A)** Statistical parametric map between the NCH and HC groups, significantly higher ReHo values are represented by warm hues and significantly lower ReHo values by cool hues. **(B)** 3D graphic representation of the ReHo results between the two groups. **(C,D)** Violin plots showing the distribution of ReHo values between the two groups in the left MPFC. **(E)** Negative correlation between MMSE scores and ReHo value in the left MPFC within the patient group (*r* = −0.5622, *p* = 0.0234; cluster-level, *p*-value <0.05, FDR corrected).

Significant differences in ReHo between the two groups, with MNI coordinates, are shown in Table 2 and Figure 2. Compared to the HC group, the NCH group showed significantly increased ReHo values in the left MPFC (cluster size = 86 voxels, *t* = 4.4991) and decreased values in the left postcentral gyrus and precentral gyrus (cluster size = 92 voxels, *t* = −4.5399), as shown in Table 2 and Figure 2. The correlation analysis revealed a negative correlation between MMSE scores and aberrant ReHo values in the left MPFC in the NCH group (*r* = −0.562, *p* = 0.023; Figure 2). No significant correlation was observed between MMSE scores and ReHo values in left postcentral and precentral gyri.

**Table 2:** Comparison of REHO values between NCH patients and HCs.

Brain area (AAL)	Peak MNI coordinates			Cluster voxel	BA	L/R/B	*t*-value
	*x*	*y*	*z*				
**NCH > HC**							
Frontal_Sup_Medial (MPFC)	−6	66	3	86	10	L	4.4991
**NCH < HC**							
Postcentral/precentral	−60	−9	18	92	—	L	−4.5399

MNI, Montreal Neurological Institute; BA, Brodmann area. (*x*, *y*, *z*), coordinates of the peak location in the MNI space, *t*-values, and statistical values of the peak voxel.

### 3.3 DC and correlation analysis

**Figure 3:**
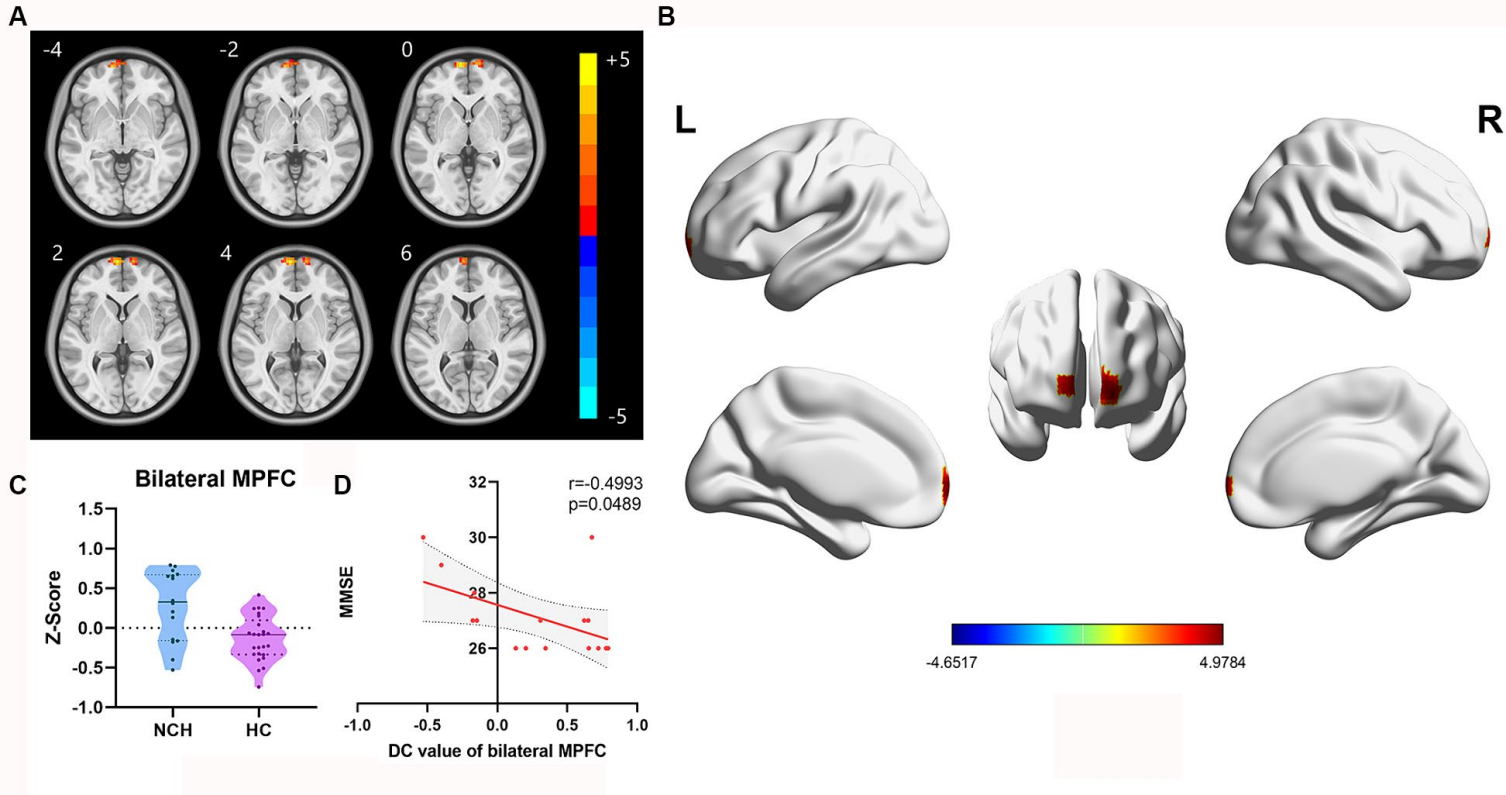
Significantly different DC map and the correlation between MMSE scores and DC value of bilateral MPFC. **(A)** Statistical parametric map between the NCH and HC groups, significantly higher DC values are represented by warm hues and significantly lower DC values by cool hues. **(B)** 3D graphic representation of the DC result between the two groups. **(C)** Violin plot showing the distribution of DC values between the two groups in the bilateral MPFC. **(D)** Negative correlation between MMSE scores and DC value in the bilateral MPFC within the patient group (*r* = −0.4993, *p* = 0.0489; cluster-level, *p*-value <0.05, FDR corrected).

Significant differences in DC between the two groups, with MNI coordinates, are shown in Table 3 and Figure 3. In comparison to the HC group, the NCH group had significantly increased DC values in the bilateral MPFC (cluster size = 61 voxels, *t* = 4.9784), as illustrated in Table 3 and Figure 3. The correlation analysis showed a significant inverse relationship between MMSE scores and aberrant DC values in the bilateral MPFC among the NCH group (*r* = −0.562, *p* = 0.023; Figure 3).

**Table 3:** Comparison of DC values between NCH patients and HCs.

Brain area (AAL)	Peak MNI coordinates			Cluster voxel	BA	L/R/B	*t*-value
	*x*	*y*	*z*				
**NCH > HC**							
Frontal_Sup_Medial (MPFC)	−9	69	0	61	10	B	4.9784

MNI, Montreal Neurological Institute; BA, Brodmann area. (*x*, *y*, *z*) Coordinates of the peak location in the MNI space, *t*-value, and statistical value of the peak voxel.

### 3.4 DMN FC and correlation analysis

**Figure 4:**
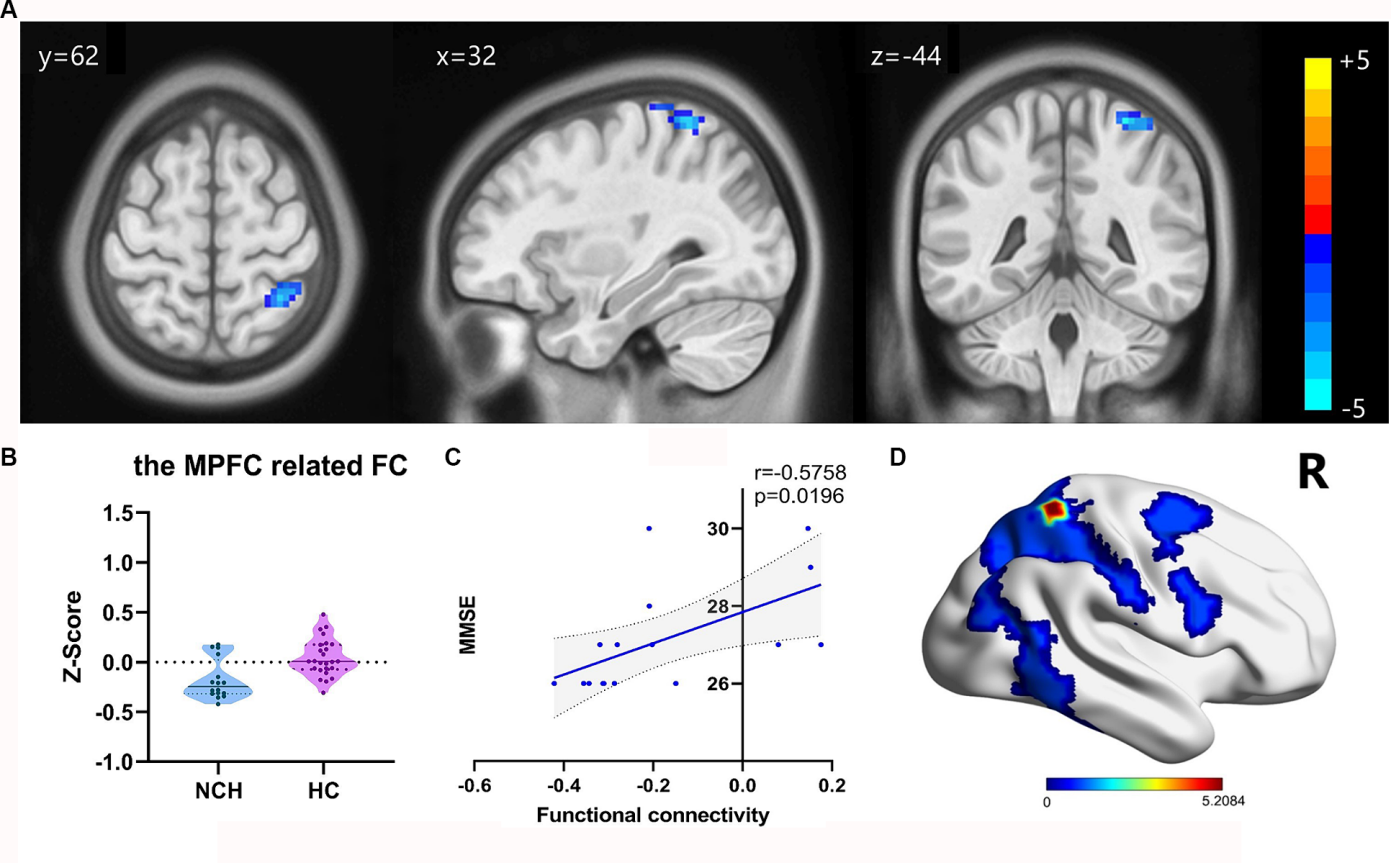
Significant differences in FC result and the correlation between MMSE scores and FC value. **(A)** Statistical parametric map between the NCH and HC groups, significantly higher FC values are represented by warm hues and significantly lower FC values by cool hues. **(B)** Violin plot of FC values distribution among the two groups. **(C)** Positive correlation between MMSE scores and FC values in the NCH group (*r* = 0.5758; *p* = 0.0196). **(D)** 3D graphic representation of the FC result between the two groups overlaid on the dorsal attention network (DAN) map based on Yeo’s et al. (34) 17-network parcellations.

We overlaid the regions of ReHo and DC values associated with the MMSE scores using an MPFC mask from the automated anatomical labelling atlas (31). These regions notably overlapped with the MPFC. Next, we analyzed the FC of the MPFC and PCC-Precuneus (Figure 1). When the MPFC was used as the ROI, the NCH group exhibited lower FC values with the right superior parietal lobe and postcentral gyrus compared to the HC group (cluster size = 70 voxels, *t* = −5.2084), as shown in Table 4 and Figure 4. With the PCC-precuneus ROI as a seed, there was no significant difference in FC between the NCH and HC groups. In the correlation analysis between DMN FC and MMSE scores, the FC value showed a significant positive correlation with MMSE scores in the right superior parietal gyrus and postcentral gyrus (*r* = 0.5758; *p* = 0.0196; Figure 4).

**Table 4:** Comparison of DMN function connectivity values between HP patients and HCs.

Brain area (AAL)	Peak MNI coordinates			Cluster voxel	BA	L/R/B	*t*-value
	*x*	*y*	*z*				
**NCH < HC**							
Parietal_Sup/postcentral	30	−48	66	70	—	R	−5.2084

MNI, Montreal Neurological Institute; BA, Brodmann area. (*x*, *y*, *z*) Coordinates of the peak location in the MNI space, *t*-value, and statistical value of the peak voxel.

## 4 Discussion

In this study, we investigated alterations in ReHo, DC, and DMN connectivity in patients with NCH and their correlations with cognitive impairment severity. As far as we know, this study represents the first exploration of regional brain activity and DMN FC in patients with NCH. A major finding was that patients with NCH exhibited increased ReHo and DC but reduced FC in the frontal core node of the DMN, all of which were associated with cognitive impairment. Specifically, In comparison to the HCs, the patients exhibited significantly higher ReHo values in the left MPFC and lower ReHo values in the left postcentral gyrus; higher ReHo values in the left MPFC were related to worse cognition. The DC values in the bilateral MPFC exhibited a significant increase in NCH patients and displayed a negative correlation with cognitive outcomes. Further, DMN FC was reduced in the right postcentral gyrus and superior parietal gyrus in patients, correlating with cognitive impairment, and alterations in ReHo were correlated with the severity of DMN functional impairment. Together, these findings suggest possible mechanisms underlying cognitive impairment in NCH and provide theoretical guidance for a better understanding of cognitive impairment in NCH.

### 4.1 ReHo

ReHo represents the local spontaneous coherence and centrality of regional neural activity. As a local functional analysis, ReHo can provide information about regional activity without *a priori* constraints and has been effectively used as a neuroimaging marker to explore brain function and abnormalities in various neuropsychiatric disorders. For example, in patients with disorders of consciousness, ReHo was found to be reduced in the PCC, MPFC, and bilateral frontal, parietal, and temporal areas but increased in the limbic system, correlating with awareness levels (13). In addition, a meta-analysis of localized connectivity studies on depression indicated robust ReHo increases in the MPFC region, associated with symptom severity (35). Furthermore, an rs-fMRI study on reading performance following tumor removal showed that reduced ReHo was related to improved reading performance (36).

Within our investigation, a notable increase in ReHo was observed in the left MPFC among patients with NCH. The MPFC, a core node of the DMN, is associated with various cognitive functions, including self-referential processing, emotional regulation, decision-making, and episodic memory (37). During periods of self-initiated episodic and social cognitive activities, the MPFC is a focal locus of neural activity (38). It also contributes significantly to the semantic and narrative aspects of speech processing and the prediction and anticipation of language understanding (39, 40). Evidence from research on Parkinson’s disease has shown that increased ReHo in the MPFCs may indicate a compensatory mechanism for preserving cognitive function. This suggests that dysfunction in the DMN plays a role in cognitive decline (41). Chen et al. (42) noted an inverse relationship between increased ReHo values in the bilateral PFC and MoCA scores, likely reflecting early-stage compensatory processes in cognitive impairment.

We observed a negative correlation between higher ReHo values in the left MPFC and MMSE scores among the patients with NCH, suggesting that enhanced local connectivity and spontaneous neural hyperactivity in the left MPFC might indicate local functional impairment due to secondary damage, such as ventricular enlargement and increased intracranial pressure, alongside potential compensatory mechanisms (14). This inverse relationship between regional brain activity coherence and cognitive function mirrors patterns reported in other disorders, where local connectivity negatively correlates with reading performance and social communication in reading difficulties in autism spectrum disorders (36, 43). We speculate that compensatory changes in the MPFC might progress alongside worsening cognitive impairment, though this mechanism requires further investigation.

Notably, patients with NCH had reduced ReHo values in the left postcentral and precentral gyri, with no significant correlation between MMSE and reduced ReHo values. These regions are core areas of the somatomotor network (SMN), crucial for somatosensory and motor cortex function, respectively. The postcentral gyrus receives peripheral somatosensory inputs, whereas the precentral gyrus is vital for motor execution and cognitive processes (44) and participates in sensorimotor integration (45). Research on Parkinson’s disease suggests that abnormal ReHo values in M1 and S1 might explain the clinical motor deficits (41). Additionally, Fabbro et al. (21) revealed reduced motor network connectivity among patients with iNPH in comparison to controls, with connectivity alterations associated with clinical symptom improvement after treatment. We suggest that the decreased ReHo in the postcentral and precentral gyri may reflect underlying motor dysfunction in patients with NCH.

### 4.2 DC

DC, a method driven by data at the voxel level, examines the strength of FC within the network of the entire brain, identifying important brain hubs with altered connectivity (46). Our findings indicated that patients with NCH showed higher DC values in the bilateral MPFC compared with the HCs, mirroring regions of higher ReHo. This suggests that in patients with NCH, the MPFC not only shows enhanced local connectivity and spontaneous neural activity but is also identified as a highly global FC hub.

A negative correlation was found between MMSE scores and increased DC values. That is, greater connectivity intensity of the MPFC as a hub is associated with greater cognitive impairment, indicating that the connectivity strength of the MPFC might increase with disease progression. This observation, along with the ReHo findings, supports the compensatory hypothesis.

In various diseases, including mild cognitive impairment (47), multiple sclerosis (48), and Alzheimer’s disease, significantly altered DC values in the MPFC indicate abnormal brain regions associated with cognitive performance. Studies on FC density changes across the whole brain have shown that increased DC in the MPFC correlates with cognitive decline, supporting the compensation mechanism in Alzheimer’s, where more local networks are adaptively integrated to offset cognitive loss as the disease progresses (49).

We suggest that the high DC value in the MPFC implies that this node contributes significantly to the entire functional network in patients with NCH and has a broad potential influence within and beyond the DMN through its connections. Although DC can reveal the number of direct functional connections, it does not indicate the directionality of these connections. Therefore, seed-based FC analysis should be performed as a complementary measure.

### 4.3 DMN FC

In the current study, FC analysis was conducted to explore the temporal correlation between the core nodes within the DMN and the entire brain. Using the MPFC as the ROI, we observed reduced DMN connectivity in the right superior parietal lobe and postcentral gyrus among the NCH group compared to the HC group. Correlation analysis further revealed a positive correlation between DMN connectivity and MMSE scores.

Fabbro et al. (21) observed a significant reduction in DMN connectivity among patients with iNPH compared to healthy individuals. Kanno et al. (50) evaluated patients with iNPH with severe cognitive impairment before and after shunt operations by analyzing DMN FC and diffusion tensor imaging indexes. The results showed lower DMN connectivity in patients than in HCs and that those with reduced DMN connectivity presented with severe memory impairments. Patients with decreased DMN connectivity have poorer cognitive and executive function scores. These observations align with our findings, suggesting that reduced DMN connectivity is associated with poor cognitive performance in patients with hydrocephalus.

In a separate large-scale brain network study (22) in iNPH, findings indicated a reduction in DMN connectivity within the patients with iNPH. The decline in DMN connectivity among patients with iNPH showed a positive correlation with the severity of iNPH symptoms and a negative correlation with cognitive dysfunction, characterized by a noticeable attention deficit. This compensatory decline in DMN connectivity may not be sustained as the patient’s symptoms worsen (22).

Hydrocephalus exerts extensive pressure throughout the brain, causing damage to multiple brain structures in the cortex and subcortex and disrupting connectivity among diverse brain areas (1). The functional and structural connectivity changes in patients with hydrocephalus involved multiple networks, including the default mode network, dorsal attention network (DAN), somatomotor network, and executive-control network, corresponding to clinical symptoms (20, 21, 51). The cognitive deficits in patients with hydrocephalus are associated with disruptions in both intra- and inter-network functional connectivity. These disconnections frequently occur within the DMN and between the DMN and the DAN (20, 52).

Even though functional MRI in NCH has not been previously documented, our findings are in line with earlier observations of hydrocephalus. Specifically, reduced connections with the MPFC were found in the right superior parietal cortex and postcentral gyrus. The right superior parietal cortex and postcentral gyrus pertain to two subnetworks of the DAN, respectively (34). These brain areas play a role in higher cognitive functions, visuospatial information processing, and control of spatial attention by linking sensory information to motor responses (53, 54). Notably, these regions may explain the cognitive impairments in the visuospatial perception, attention, and motor domains that are often observed in patients with hydrocephalus (55).

Research has shown that functional and structural alterations in the DAN significantly correlate with cognitive function (56). The DAN is highly predictive of cognitive function (57). Furthermore, a previous study indicated the significance of the connection between the postcentral gyrus and the superior parietal gyrus in the DMN, which is critical for integrating external information with internal memory (53). A recent study investigated functional network connectivity in patients with iNPH and found reduced FC between the DMN. DAN might result from impaired information exchange, contributing to deficits in attention, sensory function, and cognition, further aggravating clinical deficiencies (52).

Studies have also reported that ventricular enlargement can stretch and compress periventricular fibers, which extend from the frontal lobe to the parietal region, connecting parts of the DMN and DAN, leading to impaired cross-network FC (58, 59).

Overall, we hypothesize that damaged functional connections between specific regions within the DMN and DAN may be responsible for the pathophysiological processes of NCH with cognitive deficits. Further investigations are necessary to validate the implications of changes in the DMN and DAN to support the pathophysiology of NCH.

Our study’s ReHo and DC analyses indicated overlapping metrics in the MPFC, suggesting this region’s crucial role in NCH as a core hub of the DMN. ReHo reflects local spontaneous coherence and centrality of regional neural activity, whereas DC highlights the MPFC’s importance in the brain network architecture in NCH. Moreover, ROI-based FC analysis indicated specific regions of abnormal DMN connectivity. These metrics, depicting neuroimaging characteristics from different perspectives, complement each other and enhance understanding of the pathophysiological changes in NCH with cognitive impairment. Aberrant ReHo and DC in the MPFC, along with reduced DMN FC, are potential biomarkers for the severity of cognitive impairment in NCH.

### 4.4 Strengths and limitations

This marks the first investigation of NCH using rs-fMRI. We employed ReHo, DC, and ROI-FC analyses to explore the mechanisms of cognitive impairment in patients with NCH from multiple perspectives. However, this study has some limitations that should be considered. First, the sample size of patients included in this study was relatively small. Future studies with larger cohorts could enhance data repeatability and reliability and validate our findings. Second, our cross-sectional study was limited in its assessment of cognitive changes over the course of disease development. Future prospective studies could expand our understanding of this issue.

## 5 Conclusion

In summary, alterations in regional neural activity and FC in NCH patients were observed and closely associated with cognitive impairment. These findings contribute significantly to our understanding of the pathophysiological mechanism of NCH with cognitive impairment and hold promise as potential biomarkers for assessing the severity of cognitive impairment in NCH.

## Data availability statement

The raw data supporting the conclusions of this article will be made available by the authors, without undue reservation.

## Ethics statement

The studies involving humans were approved by the Ethics Committees of Beijing Tiantan Hospital and the People’s Hospital of Xinjiang Uygur Autonomous Region. The studies were conducted in accordance with the local legislation and institutional requirements. The participants provided their written informed consent to participate in this study.

## Author contributions

XH: Conceptualization, Methodology, Validation, Visualization, Writing – original draft, Writing – review & editing. LJ: Conceptualization, Data curation, Methodology, Visualization, Writing – original draft. TC: Data curation, Investigation, Validation, Visualization, Writing – original draft. JiaL: Data curation, Investigation, Project administration, Writing – review & editing. YQ: Data curation, Methodology, Writing – original draft. JinL: Data curation, Formal analysis, Writing – review & editing. WB: Data curation, Formal analysis, Validation, Writing – original draft. CL: Conceptualization, Funding acquisition, Data curation, Project administration, Writing – review & editing. JW: Methodology, Conceptualization, Funding acquisition, Resources, Supervision, Writing – review & editing.
